# Preoperative Determination of Depth of Invasion in Oral Cavity Squamous Cell Carcinoma by Standard Cross-Sectional Imaging With Computed Tomography and Positron Emission Tomography/Computed Tomography

**DOI:** 10.7759/cureus.40794

**Published:** 2023-06-22

**Authors:** Kushal Naha, Gregory Biedermann, Ayman Nada, Joseph Cousins, Lester Layfield, Jennifer Schnabel

**Affiliations:** 1 Hematology and Medical Oncology, University of Missouri, Columbia, USA; 2 Radiation Oncology, University of Missouri, Columbia, USA; 3 Radiology, University of Missouri, Columbia, USA; 4 Pathology and Laboratory Medicine, University of Missouri, Columbia, USA; 5 Medical Research Office, University of Missouri, Columbia, USA

**Keywords:** positron emission tomography, computed tomography, preoperative period, depth of invasion, head and neck cancer, oral squamous cell carcinoma

## Abstract

Background

Depth of invasion (DOI) is a known indicator of metastatic potential in oral cavity squamous cell carcinoma (SCC). Our purpose was to investigate the accuracy of preoperative determination of DOI in oral cavity SCC by computed tomography (CT) and positron emission tomography/computed tomography (PET/CT).

Methodology

A retrospective study was performed using consecutive patients with histologically proven oral cavity SCC presenting to our otorhinolaryngology department between January 2014 and July 2019 who underwent preoperative contrast-enhanced CT and/or PET/CT. Pathological assessment of DOI was determined by a review of pathology reports. The degree of DOI determined by radiographic studies was correlated to pathology results.

Results

A total of 79 patients were screened of whom appropriate radiographic studies were available for 63 patients. The mean DOI by pathological assessment was 12.3 ± 9.1 mm. CT and PET/CT determined depth both correlated with pathological DOI (r = 0.710; p < 0.001, r = 0.798; p < 0.001). No significant correlation was seen for CT-determined depth (r = 0.136; p = 0.709) or PET-determined depth (r = 0.234; p = 0.707) with pathologically confirmed superficial tumors (<5 mm). For patients with pathological tumor depth >10 mm, CT and PET determined depth both correlated with pathological depth (r = 0.577; p = 0.002, r = 0.668; p = 0.001). The sensitivity and specificity of CT and PET for the identification of deep invasion were 88.2% and 41.7% and 52.9% and 50%, respectively.

Conclusions

DOI measurement is feasible with routine preoperative CT and PET/CT images and is comparable to pathological measurement in patients with oral cavity SCC.

## Introduction

Cancers of the oral cavity and pharynx constitute the eighth leading cause of cancer among US males [1]. It is estimated that over 50,000 new cases of oral cavity cancer occurred in 2020 with >10,000 oral cancer-related deaths. Worldwide, the prevalence of oral cancer is even higher, with an estimated annual incidence of >350,000 and almost half as many deaths [2]. The vast majority of these cases show squamous histology. Squamous cell carcinoma (SCC) of the oral cavity is, therefore, considered synonymous with oral cancers. Surgical resection is the treatment of choice. However, locally advanced disease requires a trimodal approach involving adjuvant chemoradiation. Therefore, accurate preoperative staging is imperative for the determination of the optimal therapeutic approach [3].

The traditional staging of oral cavity cancers by the American Joint Committee on Cancer (AJCC) system reflects the importance of nodal involvement in determining prognosis. The AJCC eighth edition further refines this system by including the depth of invasion (DOI) as an important component of T staging [4]. This subtle but important distinction rests on the current understanding that DOI within the primary tumor is closely linked with occult nodal involvement and thereby the overall prognosis of these tumors [5-7] and has been retrospectively validated by comparison with the AJCC seventh edition [8].

The presence of clinically evident cervical lymphadenopathy in patients with oral cancer mandates neck dissection regardless of the presence or absence of primary tumor depth. On the other hand, the decision to proceed with elective lymph node dissection in patients with clinically node-negative disease is more nuanced. Unfortunately, even modern imaging techniques such as positron emission tomography/computed tomography (PET/CT) are relatively limited in their ability to detect clinically occult lymph node involvement [9]. This realization has led to the exploration of DOI as a surrogate for occult node involvement. Although considered the gold standard, pathological estimation of DOI can be challenging because of the need for rapid assessment of intraoperative frozen tissue specimens and concerns about tumor shrinkage during processing leading to underestimation of DOI. Moreover, this approach negates the possibility of preoperative treatment planning or the use of neoadjuvant therapy in selected individuals. Dependence on intraoperative pathological evaluation can also lead to delays in surgery or even inappropriate application of elective node dissection. These shortcomings have spurred the development of radiographic techniques for the non-invasive assessment of DOI [10].

Of the conventional modalities available, magnetic resonance imaging (MRI) has been most extensively evaluated as a tool for the assessment of DOI based on its inherent superiority for soft tissue imaging and freedom from artifacts from adjacent bone or metallic dental implants. MRI-determined DOI has been directly correlated with the presence of clinically occult nodal involvement, locoregional control, and disease-specific survival [11].

Computed tomography (CT) is widely employed in preoperative surgical planning for the determination of tumor anatomy and its relationship to adjacent structures, largely due to advantages in availability, cost, speed, and ease of performance [12]. Likewise, PET is broadly employed for the detection of nodal and distant metastases in patients with head and neck cancers [13,14]. Although at a disadvantage compared to MRI because of limitations imposed by image resolution and bone and metal artifacts, CT and PET carry advantages in patient comfort and convenience and are less susceptible to motion artifacts [15]. Moreover, accurate determination of DOI and other primary tumor characteristics by CT and PET would obviate the need for separate MRIs and ultimately lower healthcare costs.

This study was designed to determine the accuracy of preoperative determination of DOI in oral cavity SCC by standard cross-sectional imaging with CT and fused PET/CT by comparison with the gold-standard pathological measurement in the resected surgical specimen.

## Materials and methods

Study settings

The University of Missouri at Columbia is a major tertiary care and referral center in Missouri, providing care to the surrounding region of mid-Missouri with an estimated population of over 400,000 individuals.

Patients

**Figure 1:**
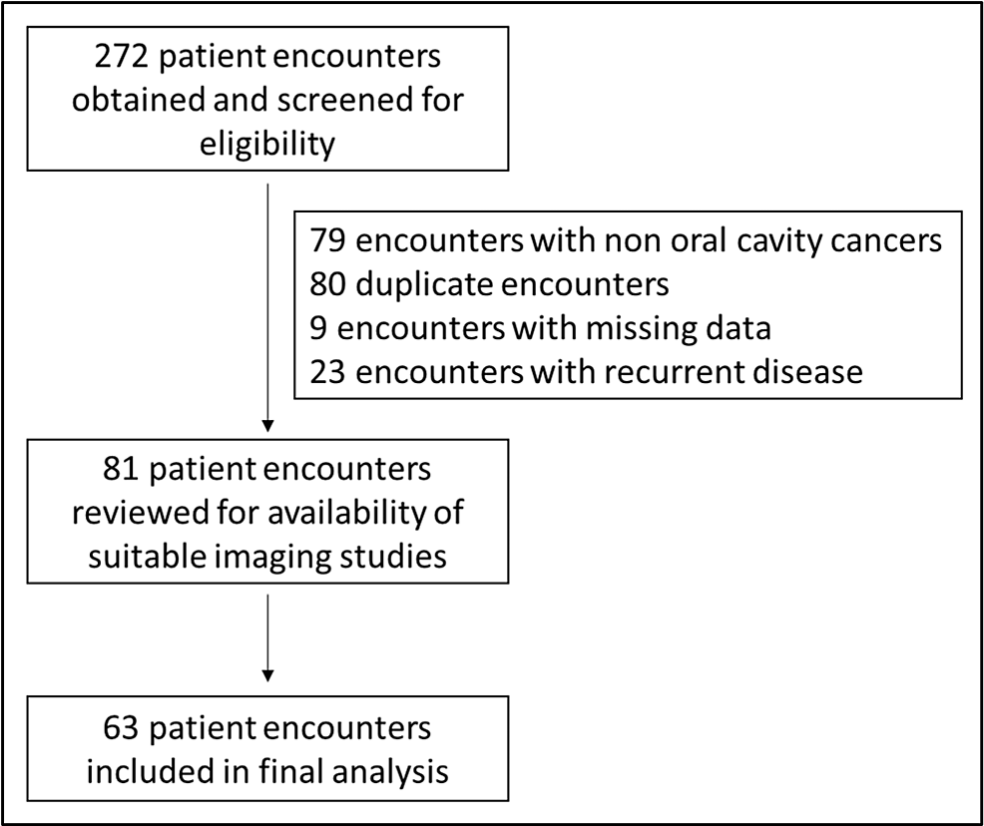
Flowchart showing the screening process and selection of patients for the study.

Institutional IRB approval was obtained for performing a retrospective record-based study with a consent waiver (approval number: 2004777). Consecutive patients with histologically proven oral cavity SCC presenting to the otorhinolaryngology department between January 2014 and July 2019 were included in the study. All patients underwent preoperative imaging with contrast-enhanced CT of the neck and/or PET/CT. Patients for whom such studies were not available in the prerequisite resolution and those for whom pathological data on DOI were unavailable were excluded from the study. Patients with recurrent oral cavity SCC were also excluded because of the possibility of radiologic distortion arising from previous surgeries. This process is summarized in Figure 1.

Imaging protocol

CT Protocol

High-resolution contrast-enhanced CT of the neck was performed with a slice thickness of 1.25 mm, tube potential of 100 kV, tube current of 223-247 mA, pitch factor of 0.8, and matrix of 512 × 512. Approximately 70-120 mL of intravenous contrast medium (Omnipaque (Iohexol) 300 mg, GE Healthcare Inc, Boston, MA, USA) was administered with an injection rate of 2-3 mL/second. Images were reformatted into 3 mm slice thickness in axial, coronal, and sagittal planes for interpretation. Axial 3 mm images of the bone algorithm were also reformatted for evaluation of bony invasion, i.e., mandible or maxilla.

PET/CT Protocol

PET/CT studies were acquired on a PET/CT scanner (Siemens Biograph mCT 64, Siemens Healthineers, USA). Patients were instructed to fast for at least four to six hours with a preferred glucose level of 60-120 mg/dL before intravenous injection of 3-15 mCi FDG (0.04-0.088 mCi/kg) for image acquisition. PET emission data were acquired with a two-minute emission time per bed position from the vertex of the skull to the proximal thighs. Consecutively, transmission data were acquired using low-dose non-contrast-enhanced CT with 5 mm slice thickness and 780 mm field of view. Post-processing iterative reconstruction of PET data was performed with two iterations, 21 subsets, and Gaussian filtering with attenuation correction.

Imaging evaluation and measurement of the DOI

Radiographic studies were independently reviewed by two radiologists (with 15 and five years of experience in head and neck radiology) who were blinded to the results of the pathological assessment.

Tumor Site

The site of primary oral cavity SCC was determined and assigned to the oral cavity subsites, e.g., the oral tongue (anterior and lateral aspects of the tongue) and the floor of the mouth.

Locoregional Involvement

The extension of the primary tumor, invasion of the extrinsic muscle of the tongue, and mandibular invasion were evaluated. Cervical lymph nodes were evaluated for involvement based on radiologic criteria. The lymph node station, size, number, side of lymph nodes, as well as the presence of extranodal extension, were determined in cases with detectable lymphadenopathy. Occult lymphadenopathy was determined in cases where imaging findings were negative for lymphadenopathy but pathological evaluation showed lymph node involvement with cancer.

Distant Metastasis

Distant metastasis is rare in the case of oral cavity SCC. Hence, a metastatic workup is not routinely done for these patients. We were able to evaluate distant metastasis in patients who underwent PET/CT for their evaluation. The site of distant metastatic deposits was recorded, e.g., lung, bone, or brain.

Staging according to AJCC eighth edition

Findings from imaging studies as noted above were combined using the TNM staging system to determine the stage according to the AJCC eighth edition [4]. Accordingly, 16 patients were in stage I, 14 patients were in stage II, 14 patients were in stage III, 17 patients were in stage IVA, and two patients were in stage IVB. There were no patients with IVC disease as none of the included patients had evidence of distant metastases.

Measurement of DOI and Correlation to Pathology Results

Anteroposterior measurements were used for ventral lesions while transverse measurements were used for lateral tongue lesions. Radiographic assessment of DOI was compared with the results of pathology. Cases were subdivided into superficial and deep categories for the purpose of subset analysis defined as pathological DOI of less than 5 mm and greater than 10 mm, respectively.

Pathological analysis

Pathological assessment of DOI in the tumor specimen was separately determined by a review of pathology reports. All included patients underwent presurgical biopsy followed by surgical resection. Only surgical resection specimens were used for the assessment of DOI to ensure optimal representation of the tumor. Specimen slides were reviewed by the pathologist to ensure accurate measurement of DOI.

Statistical analysis

All data were analyzed using SPSS software version 25.0 (IBM Corp., Armonk, NY, USA). Continuous variables were presented as mean ± standard deviation (SD). The correlation between radiographic DOI and pathological DOI was determined using linear regression analysis. The sensitivity and specificity of CT and PET/CT for the detection of deep invasion were calculated using 2 × 2 contingency tables. A p-value of less than 0.05 was considered statistically significant.

## Results

A total of 79 patients with oral cavity SCC were screened for the study, of whom 63 had undergone appropriate radiographic assessment and were included in the final analysis. Of these individuals, CT images were available in 55 cases while PET/CT images were available in 33 cases.

Patient demographics

The mean age of included individuals was 61.5 ± 13.3 years. The majority were male (71.4%), of Caucasian ethnicity (96.8%), and were either current or former smokers (84.1%).

Imaging evaluation

**Figure 2:**
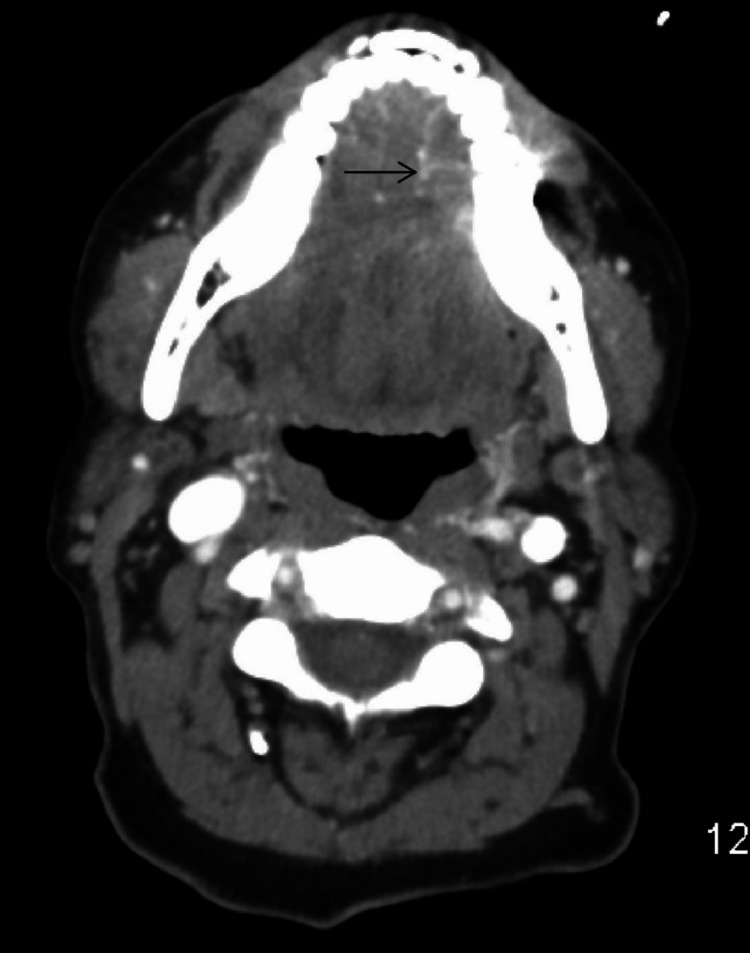
A 57-year-old female with moderately differentiated oral squamous cell carcinoma. Axial contrast-enhanced CT image demonstrates heterogeneously enhancing mass in the left lateral tongue with peripheral enhancing capsule and mild medial displacement of the neurovascular bundle. CT: computed tomography

**Figure 3:**
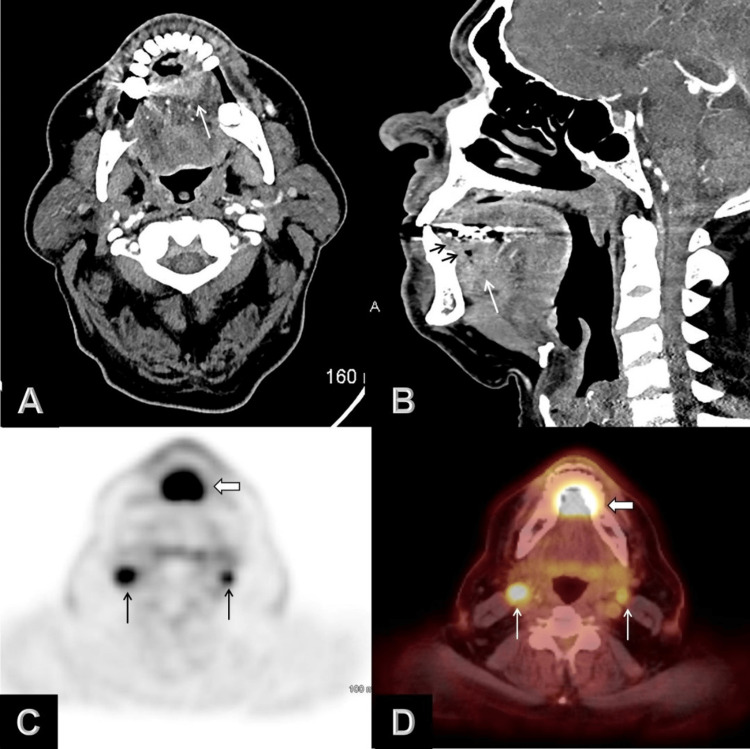
Axial (A) and sagittal (B) contrast-enhanced CT images show an ulcerative mass (white arrows) involving the floor of the mouth and anterior midline ventral aspect of the tongue with infiltration of the genioglossus muscle. The mass infiltrates the gingival mucosal lining of the lingual mandibular cortex (black arrows). Axial 18F-FDG PET (C), and axial fused PET/CT (D) images demonstrate the floor of the mouth mass with avid FDG uptake (wide arrows) and bilateral level 2 FDG-avid lymph nodes (arrows). CT: computed tomography; PET: positron emission tomography; FDG: fludeoxyglucose

Nodal involvement was seen in 24 cases, while clinically evident metastatic disease was not seen in any of the included patients. Baseline patient characteristics are summarized in Table 1. Representative examples of imaging from our patients are shown in Figure 2 and Figure 3.

**Table 1:** Baseline patient characteristics. AJCC: American Joint Committee on Cancer

Characteristic	N = 63
Age (mean ± SD)	61.5 ± 13.3 years
Sex	
Male	71.4%
Female	28.6%
Ethnicity	
Caucasian	61 (96.8%)
Others	2 (3.2%)
History of smoking	53 (84.1%)
T staging (AJCC v8)	
pT1	19 (30.2%)
pT2	25 (39.7%)
pT3	10 (15.9%)
pT4a	7 (11.1%)
pT4b	2 (3.2%)
N staging (AJCC v8)	
pN0	39 (61.9%)
pN1	9 (14.3%)
pN2 (a/b/c)	14 (22.2%)
pNx	1 (1.6%)
Pathological depth of invasion (mean ± SD)	12.3 ± 9.1 mm
Depth of invasion <5 mm	12 (19.0%)
Depth of invasion 5–10 mm	20 (31.7%)
Depth of invasion >10 mm	31 (49.2%)

Pathological evaluation of DOI

The most common pathologic T stage was pT2 seen in 39.7% of cases, and the mean pathological DOI was 12.3 ± 9.1 mm. Deep tumors were present in 49.2% of cases.

**Figure 4:**
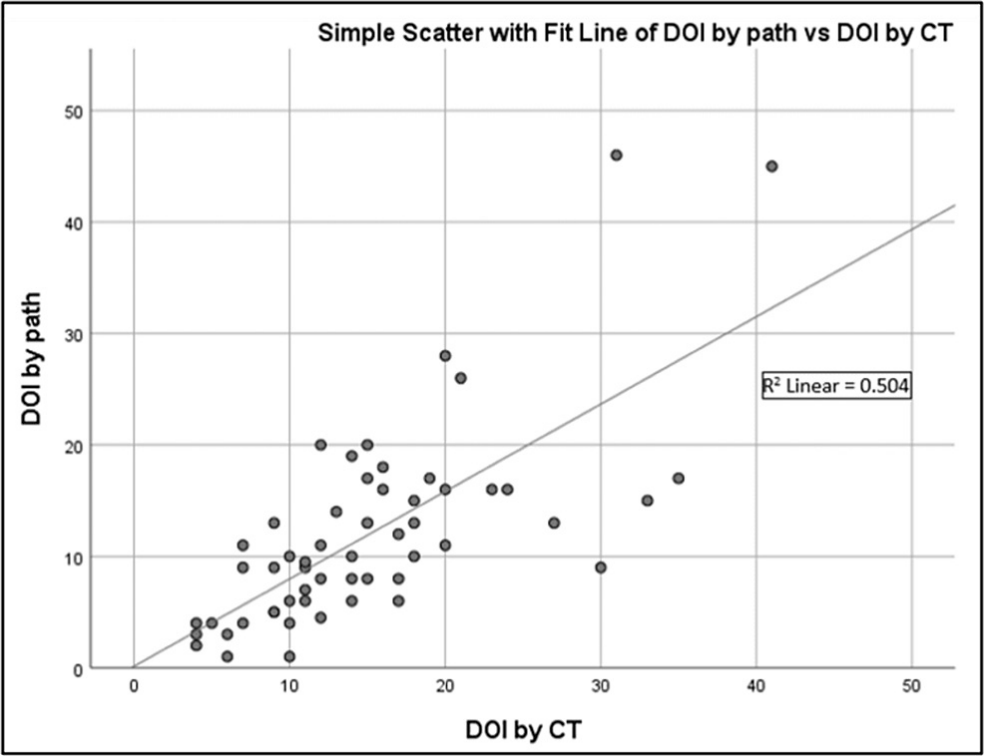
Simple scatter plot showing the correlation between DOI by pathologic examination and DOI by CT. DOI: depth of invasion; CT: computed tomography

**Figure 5:**
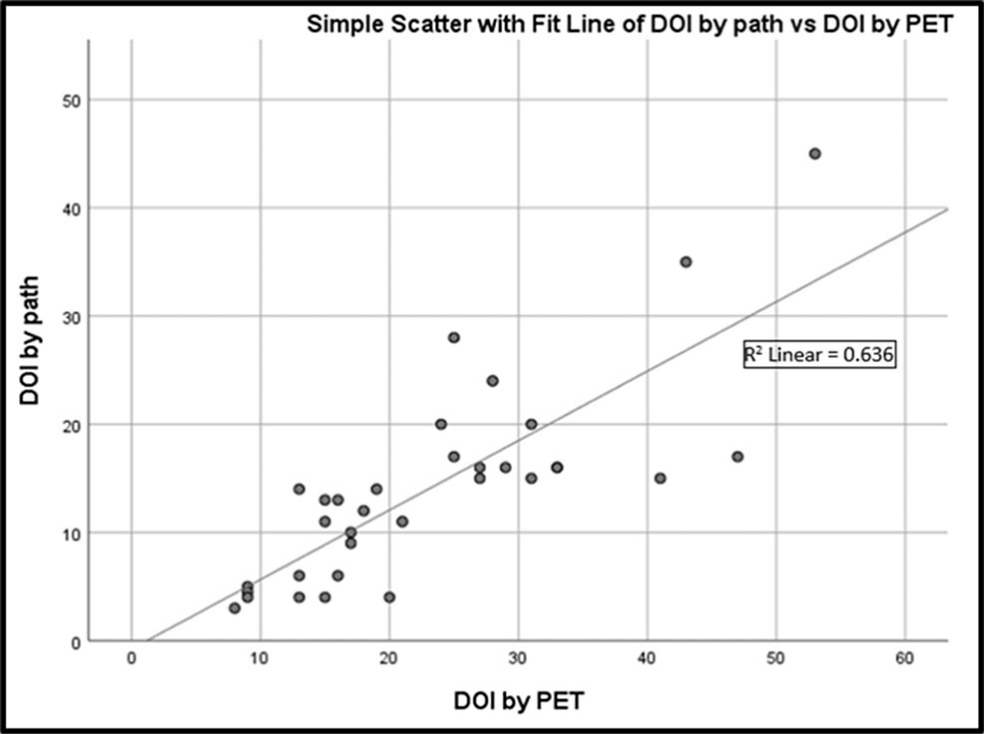
Simple scatter plot showing the correlation between DOI by pathologic examination and DOI by PET. DOI: depth of invasion; PET: positron emission tomography

**Figure 6:**
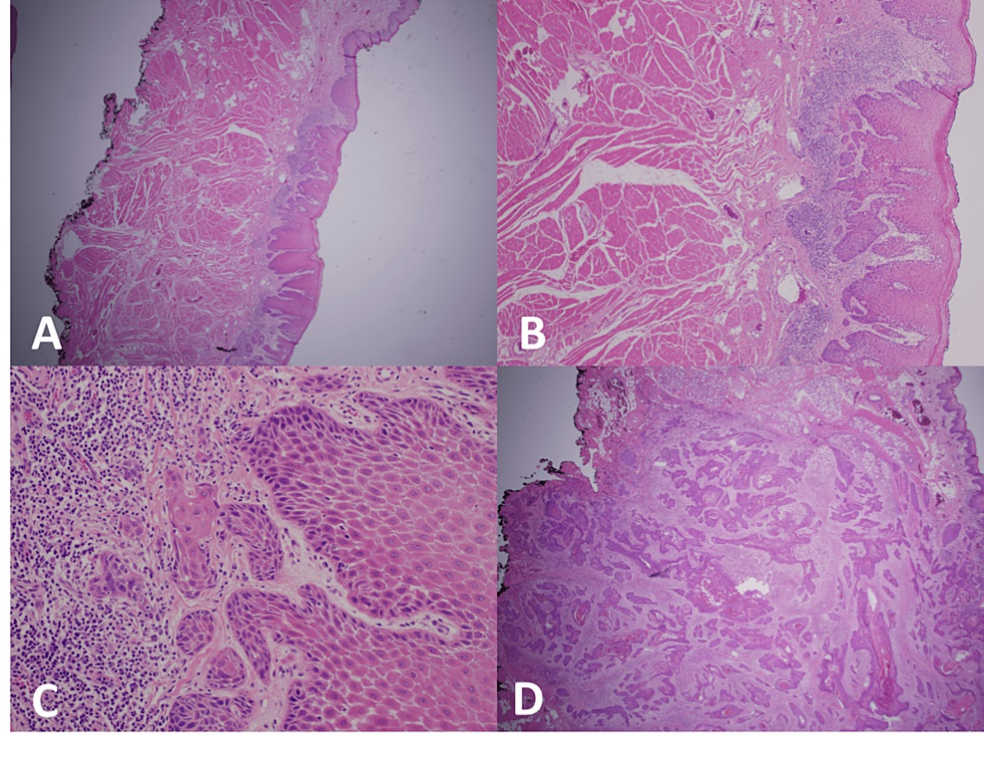
Pathology specimen showing invasive squamous cell carcinoma with a depth of invasion of 1 mm, hematoxylin and eosin staining (H&E) ×12.5 (panel A); H&E ×20 (panel B); H&E ×100 (panel C); invasive squamous cell carcinoma with a depth of invasion of 16 mm, H&E ×12.5 (panel D).

CT-determined depth (r = 0.710; p < 0.001) and PET/CT-determined depth (r = 0.798; p < 0.001) both correlated well with pathological DOI. Sensitivity and specificity for detection of deep invasion (greater than 10 mm) was 88.2% and 41.7%, respectively, for CT and 52.9% and 50%, respectively, for PET/CT. Both CT and PET/CT overestimated DOI but this tendency was more pronounced with PET (55% vs. 27%) (Figures 4, 5). Representative pathology micrographs are also provided in Figure 6.

A subset analysis was also performed to determine if the accuracy of the imaging modalities was comparable across superficial and deep tumors defined as a pathological DOI of less than 5 mm and greater than 10 mm, respectively.

Results of the subset analysis showed that CT and PET/CT were both predictive (r = 0.577, p = 0.002; r = 0.668, p = 0.001) of pathological DOI in patients with histologically confirmed deep tumors (DOI greater than 10 mm). Conversely, neither modality was predictive of pathological DOI (r = 0.136, p = 0.709; r = 0.234, p = 0.707) in superficial tumors (DOI less than 5 mm).

## Discussion

The results of our study show that preoperative CT and PET/CT imaging is a reasonable option for the determination of tumor DOI. The correlation seen in our study between pathological and radiographic estimates of tumor DOI is also impressive when the limitations of these modalities are considered vis-à-vis the more traditional option of MRI. Evaluation of CT as a preoperative tool in these settings has been previously explored in other studies. A recent Japanese study performed a head-to-head analysis of CT and MRI for the determination of DOI and demonstrated comparable accuracy between the two modalities [16]. Similarly, an Italian study evaluating CT-based DOI measurements against pathological data found this radiographic modality to be a reliable and reproducible indicator of pathological DOI, provided standardized measurement protocols were implemented [17]. The authors proceeded to suggest specific axes for the measurement of tumors in different locations within the oral cavity. This approach was similar to our own, wherein prespecified axes were used to calculate DOI based on the anatomic location of the tumor.

On the other hand, despite widespread recognition of its utility in patients with oral cavity SCC, the application of PET/CT for the assessment of DOI remains unproven in the absence of corroborative clinical data. This is largely due to known limitations in image resolution that impede accurate measurement of smaller lesions most often encountered in the oral cavity. Other drawbacks include interference from inflammatory changes induced by biopsies and non-specific uptake in the tongue related to intrinsic muscle activity and differences in muscle tone [14,18].

Our decision to include PET/CT in this study despite these perceived disadvantages was based on the fact that this modality is already widely employed in oral cavity SCC based on its effectiveness in detecting nodal involvement [19], as well as its superiority to CT and MRI in this regard [20]. PET/CT is also useful in measuring tumor volume [21], detecting the presence of bone invasion [13,22], and assessing the response to neoadjuvant and definitive chemoradiation [23], all of which suggest that it could be used to assess DOI. Moreover, PET/CT carries the added advantages of not being affected by dental artifacts [21], movement artifacts, and streak artifacts [14].

Recognizing a relative deficiency of studies investigating CT and PET/CT in this role, we also compared our results with studies on preoperative MRI assessment of DOI. Traditional MRI with a slice thickness of 4 mm is accurate in identifying tumors with DOI greater than 5 mm [24]. More superficial tumors require higher resolution MRI. Studies employing high-resolution MRI with a slice thickness of 2 mm [25] and 1 mm [26] demonstrated accuracy and reproducibility in oral SCC regardless of DOI. Interestingly, analogous to our own findings, MRI appears to overestimate DOI consistently. Tumor specimen shrinkage due to formalin fixation has been suggested as a potential mechanism to explain this phenomenon [27]. If this hypothesis holds true, then the radiographic estimation of DOI could be superior to pathological measurement. This would explain the relatively poor specificity for the detection of deep invasion by CT and PET/CT seen in our study.

Our subset analysis of CT and PET/CT in superficial and deep tumors was based on previous observations that a tumor depth of less than 5 mm is associated with a low incidence of nodal metastasis [28], while a DOI of greater than 10 mm is strongly associated with nodal involvement and increased risk of local recurrence [29]. This pattern reflects increased access of the primary tumor to local lymphatic and vascular channels with correspondingly greater DOI and a greater propensity for locoregional tumor dissemination. The negative impact of increasing DOI on clinical prognosis in patients with oral SCC is a direct consequence of this association with nodal metastasis. Indeed, a DOI greater than 5 mm has been proposed as an objective indication for elective node dissection [30]. Consequently, we were interested to see how these imaging modalities would perform with these high-risk patients. Our finding that both CT and PET/CT performed better with deep tumors is both intuitive and reassuring. The relatively small DOI in superficial tumors entails a greater margin of error leading to a weaker correlation with pathological DOI. However, the real benefit of preoperative imaging lies in the category of high-risk deep tumors, and we have shown that both CT and PET/CT performed adequately in this group.

Our study raises practical questions on the timing of preoperative imaging modalities in patients with oral cavity SCC. Of particular concern was the impact of biopsy-related changes on the radiographic estimation of DOI. Unfortunately, current insurance regulations dictate that imaging with PET/CT be performed only after tissue confirmation of cancer. Moreover, providers often choose to obtain biopsies first before embarking on imaging studies for the completion of staging and surgical planning. As it is unlikely that such conventional practices will be changed in the immediate future, it is our hope that continued improvements in the precision of radiographic techniques will be able to compensate for these problems. Ultimately, we foresee routine integration of these studies into preoperative surgical planning and decision-making on sentinel lymph node biopsy and elective neck dissection.

Although we looked for a correlation in our study between radiographic DOI and nodal involvement, we were unable to demonstrate such a relationship. We believe that this was because the study was underpowered to detect such differences, as there were only five patients with occult nodal involvement. Likewise, we were unable to show any correlation between DOI and mortality in this study because of the low rate of mortality in our patients. Other limitations of this study include its small sample size, the retrospective design, and limited applicability to non-white US adults.

## Conclusions

Despite significant technological advances in CT and PET/CT imaging, these modalities remain underutilized in the assessment of DOI in patients with oral cavity SCC. Studies in non-US populations employing CT-based measurements have shown promising results, while PET-based measurements are further hampered by a lack of supportive clinical data. Our study shows that measurements derived from routine preoperative CT and PET/CT images can be used to predict pathological DOI in these patients. Larger prospective trials are needed for the validation of these findings and evaluation of the role of these modalities in determining the need for sentinel lymph node biopsy and elective neck dissection.
